# Application of deep learning in isolated tooth identification

**DOI:** 10.1186/s12903-024-04274-x

**Published:** 2024-05-09

**Authors:** Meng-Xun Li, Zhi-Wei Wang, Xin-Ran Chen, Gui-Song Xia, Yong Zheng, Cui Huang, Zhi Li

**Affiliations:** 1 State Key Laboratory of Oral & Maxillofacial Reconstruction and Regeneration, Key Laboratory of Oral Biomedicine Ministry of Education, Hubei Key Laboratory of Stomatology, School & Hospital of Stomatology, Wuhan University, Wuhan, China; 2 Department of Oral and Maxillofacial Surgery, School and Hospital of Stomatology, Wuhan University, Wuhan, China; 3 Department of Prosthodontics, School and Hospital of Stomatology, Wuhan University, Wuhan, China; 4 School of Computer Science, Wuhan University, Wuhan, China; 5 Department of Anatomy and Embryology, School of Basic Medical Sciences), Wuhan University TaiKang Medical School, Wuhan University, Wuhan, China

**Keywords:** Artificial intelligence, Deep learning, Image classification, Image set classification, Tooth identification

## Abstract

**Background:**

Teeth identification has a pivotal role in the dental curriculum and provides one of the important foundations of clinical practice. Accurately identifying teeth is a vital aspect of dental education and clinical practice, but can be challenging due to the anatomical similarities between categories. In this study, we aim to explore the possibility of using a deep learning model to classify isolated tooth by a set of photographs.

**Methods:**

A collection of 5,100 photographs from 850 isolated human tooth specimens were assembled to serve as the dataset for this study. Each tooth was carefully labeled during the data collection phase through direct observation. We developed a deep learning model that incorporates the state-of-the-art feature extractor and attention mechanism to classify each tooth based on a set of 6 photographs captured from multiple angles. To increase the validity of model evaluation, a voting-based strategy was applied to refine the test set to generate a more reliable label, and the model was evaluated under different types of classification granularities.

**Results:**

This deep learning model achieved top-3 accuracies of over 90% in all classification types, with an average AUC of 0.95. The Cohen’s Kappa demonstrated good agreement between model prediction and the test set.

**Conclusions:**

This deep learning model can achieve performance comparable to that of human experts and has the potential to become a valuable tool for dental education and various applications in accurately identifying isolated tooth.

**Supplementary Information:**

The online version contains supplementary material available at 10.1186/s12903-024-04274-x.

## Background

The identification of isolated human tooth has always been a focus of the dental curriculum. Identification of tooth by its morphology is compulsory knowledge required for each dentistry students [1]. Tooth morphological identification is also important in almost all clinical procedures [2] and is essential throughout the whole dental career [3]. In addition, the identification of isolated human tooth has many applications in forensic odontology and archaeology. In many serious situations such as fire accidents, plane crashes, and natural disasters, or cases of corpse decomposition and disfigurement, traditional methods such as fingerprints and iris scans often fail to identify subjects because of their low resistance. In these cases, teeth that can tolerate high temperatures are considered the best candidates for human identification [4]. In the 2007 Thailand tsunami disaster in India, dental evidence was the primary source of identification in most of the cases, followed by fingerprints and DNA evidence. The identification rate of missing persons with dental records was significantly higher than those without [5]. Additionally, many of the existing researches which inferred age [6, 7], gender [8, 9] and ancestry [10] through teeth were based on one specific tooth. In anthropological and archaeological surveys, an isolated tooth also played important role in identification [11].

Recently, digital technologies have been wildly applied in dental clinical works, benefiting both dentists and patients. The application of digital scanners, cone beam tomography, and other technologies have been generating tremendous data, along with the rapid development of machine learning, accelerating the related researches in dentistry. Researches on artificial intelligence have been underway since the middle of the last century [12]. With the development of deep learning, machine learning models with vast amounts of hidden layers and massive training data can learn more complex features, significantly improving the accuracy of classification programs [13].

In the field of image recognition, convolutional neural networks (CNNs) are usually ideal choices with existing methods having achieved over 80% Top-1 accuracy and over 95% Top-5 accuracy on ImageNet datasets [14], while also demonstrating excellent performance on medical image analysis [15]. Nowadays, the identification of objects in the image has been applied to a variety of aspects from daily photos to microscopic images [16, 17]. There were several researches that have applied deep learning to classify teeth categories based on images, the majority of which focused on the classification of teeth in an entire dentition, such as the segmentation and classification of teeth in Cone-beam Computed Tomography (CBCT) images [18]; Identifying teeth through tooth impression model images [19] or dental panoramic x-ray images [20]. On the other side, single tooth volume images from CBCT images can also be identified using CNNs [21, 22]. However, there is currently no research identifying isolated tooth specimens by photographs.

Identifying isolated tooth based on appearance can be challenging due to the anatomical similarities between categories, requiring a high level of expertise. This study aims to explore the possibility of using a deep learning model to classify isolated tooth by photographs. A dataset consisting of photographs from isolated human tooth specimens were collected for this study. A CNN model was trained and evaluated on this dataset, the model was developed to take input of a set of 6 images photographed from different directions of a single tooth, and automatically predicts its category. The trained model was assessed under different classification granularities and exhibited classification accuracy comparable to that of human experts. To our best knowledge, this is the first study that classifies isolated tooth through natural photographs.

## Methods

### Sample collection

**Fig. 1:**
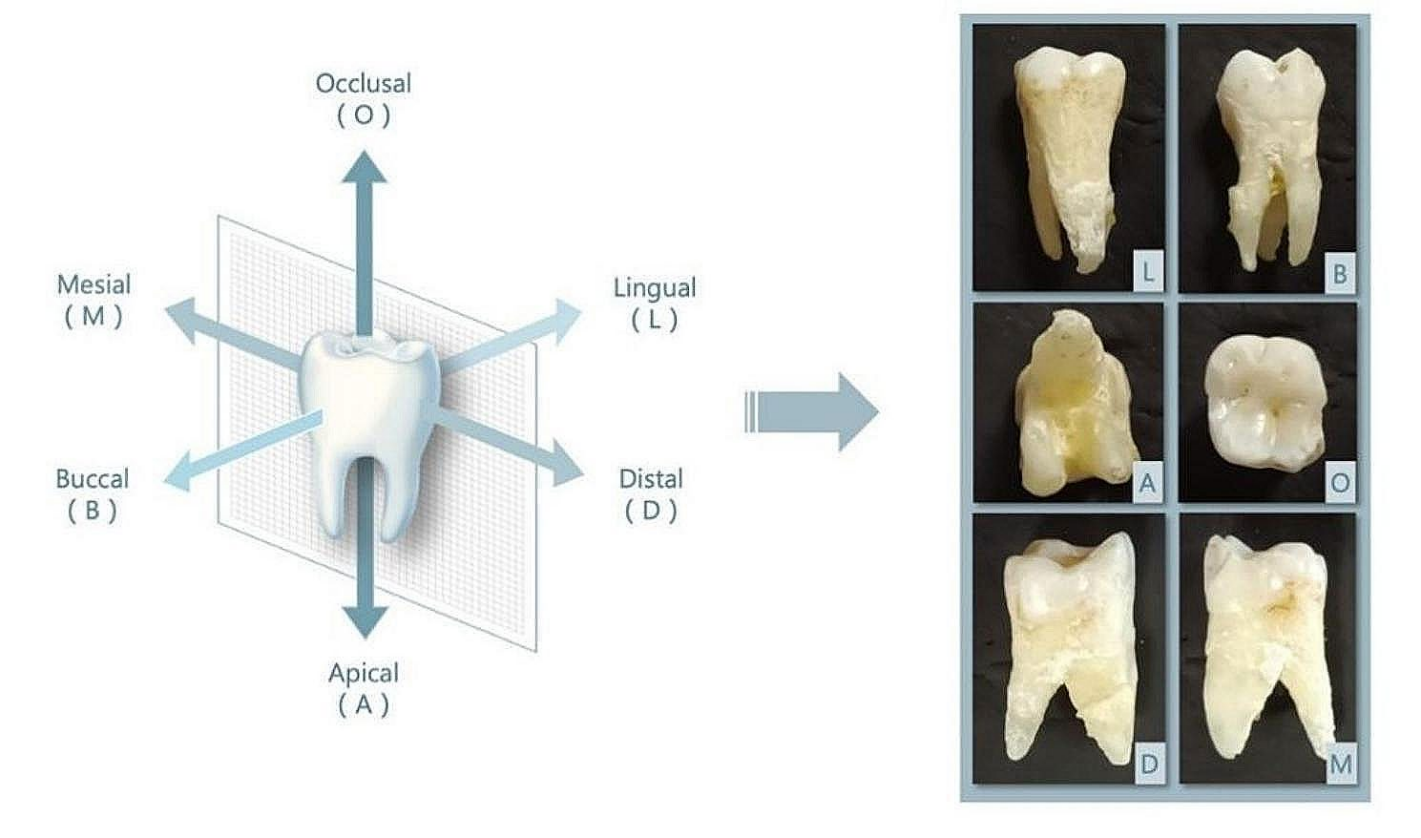
Data acquisition schematic: the tooth was photographed from six directions

A total of 850 teeth were collected from the Department of Anatomy, TaiKang Medical School of Wuhan University. The teeth included incisors, canines, premolars and molars of the maxilla and mandible. The collected teeth were photographed from six directions, specifically, occlusal, apical, buccal, lingual, mesial, and distal, respectively, as shown in Fig. 1 (Camera model: Canon Power Shot G12). Two well-trained observers, who have accomplished dental anatomy courses and have one year of clinical experience, classified the 850 teeth based on the tooth anatomy characteristics. The teeth were labeled into 16 categories, namely, maxillary 1–8 and mandibular 1–8 (according to FDI tooth classification, without distinguishing between left and right). To minimize data annotation errors, the following measures were taken: the two observers strictly compared the morphology of sample tooth to the standard, every tooth was identified by the two observers, separately. In the cases when they didn’t reach an agreement, a third observer was consulted. The third observer is an experienced teacher, who has more than 30 years of teaching experience on tooth anatomy. Data were valid only if the three observers reached an agreement.

### Dataset

A total of 5,100 images from the 850 teeth were collected to serve as the database for this study. The photos were randomly divided into a training set of photographs from 650 teeth and a raw testing dataset (raw test set) of photographs from 200 teeth. The images from the training set were used to train the machine learning model with supervision from categories annotated by direct observation. To ensure the reliability of the labeling, the raw testing set was further refined using a voting strategy, as described in the following section, to minimize annotation error and accurately evaluate the model’s performance.

### Voting-based test set refinement

In our practice, we observed that in many circumstances, the ground truth category of a tooth was unknown because of the non-standard anatomy. The observed category in essence was a maximum likelihood estimate of the ground truth category and was not guaranteed to be correct. A more accurate label in the testing dataset would be essential to accurately reflect the model’s classification accuracy.

To this end, we employed a voting strategy to filter out ambiguous data and generate labels with more confidence by seeking agreement between different observations. After image collection, an experienced teacher (the third observer) labeled the raw test set teeth by inspecting their images using an in-house developed software to generate his annotation (A1). And the two observers who collected the image labeled the raw test set again after one year of the data collection, using the same software and generated their annotation (A2 and A3). The labeling software was designed to allow users to freely explore the 6 photographs taken from different directions of one tooth and adjust contrast and brightness to have a comprehensive view of the image set. Together with the direct observation annotation by the two observers (A0), we voted to generate the resulting label with more confidence by giving A0-A3 different weighting scores. Specifically: we gave A0, A1, A2, and A3 weighting scores of 2, 2, 1, and 1, respectively. A tooth would be labeled as a category with the highest score only if the category got a score equal to or larger than 4, otherwise, the tooth would be removed from the raw test dataset. In this way, we filtered 71 ambiguous tooth data out of the raw test set and generated the test set with more confidence which constitutes images from 129 teeth. The resulting test set was used to evaluate the model’s performance, and the designated label for the test set was referred to as the “pseudo-ground-truth” (P-GT) in the following part of this article.

### Deep learning

**Fig. 2:**
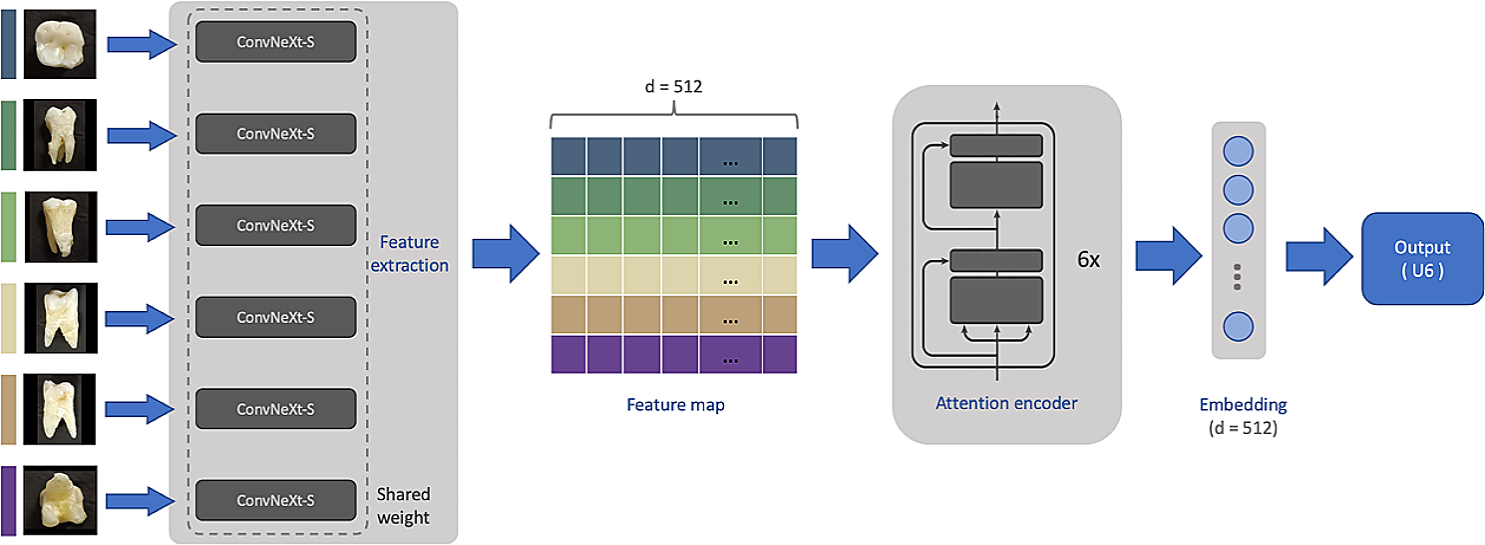
The proposed model: It consists of a feature extraction encoder and an attention encoder to generate the possibility of the image set being each category by taking an image set of 6 images from a single tooth as the input

The proposed deep learning model took an image set of 6 photographs from a single tooth as the input on each forward pass, and generated the possibility of the image set being each category. The model topology is illustrated in Fig. 2, the model mainly consists of a feature extraction encoder to extract feature of each image and an attention encoder to fuse and correlate the generated features. We adopted the ConvNeXt-S [23] as the feature extraction backbone. These features are then processed by the attention encoder, which is the same as that in the Transformer [24], to fuses and correlates the features and generate a prediction about the category of the entire image set. Specifically, the six images share the same feature encoder weights, which reduces the computational complexity, while fully utilize the capacity of the feature extractor.

To ensure that the input images are compatible with the model, they will be resized to a square shape with dimensions of 224 × 224 pixels. The resizing process will maintain the aspect ratio of the original image and pad any missing pixels on the shorter side with black color. This is important because the aspect ratio may serve and a valuable anatomical feature for teeth classification.

The model was trained using the Adam optimizer with default settings (alpha and beta parameters of 0.9 and 0.99), a weight decay factor of 0.5 was set for regularization. Additionally, run-time data augmentation by random rotations, dropout, perspective, and affine transformations were applied with probabilities of 0.5, 0.2, 0.2, 0.2, respectively, to further regularize the model.

The model was trained with a batch size of 16 teeth (96 images) for 300 epochs using cross-entropy loss. Poly learning rate with a power of 1.5 and a warmup phase of 60 epochs were used to stable the training process. To address uncertainty in human annotations, a label smoothing factor of 0.1 was added to the loss calculation to reduce the teacher-forcing problem.

The experiment was conducted on a single Nvidia RTX 3090 GPU (Nvidia Corporation, Santa Clara, CA, United States), the training time took approximately 2.5 h to complete.

### Model evaluation

Initially, the data were labeled into 16 categories during data collection phase. However, during the later stage of this study, we noticed that controversy often existed between different human observers regarding the tooth category in some images. The evaluation based on the uncertain label might be inappropriate. In addition to the voting-based test set refinement, we empirically grouped the teeth into 3 types of classifications with different granularity by merging similar sub-categories into a super-category:

- **16-type classification**: Maxillary 1–8 and mandibular 1–8 (according to FDI tooth classification). This is the same classification as the manual label. For easier illustration, we named them U1-U8 (upper jaw 1–8) and L1-L8 (lower jaw 1–8).
- **6-type classification**: Anterior teeth (1–3), premolar (4–5), molar (6–8), subcategorized into the upper and lower jaw. Namely: upper anterior teeth (UA), upper premolar (UP), upper molar (UM), lower anterior teeth (LA), lower premolar (LP), and lower molar (LM).
- **3-type classification**: Anterior teeth (1–3), pre-molar (4–5), molar (6–8), without distinguishing upper and lower jaw. Namely: anterior teeth (A), premolar (P), and molar (M).

More categories will better reflect the model’s ability in distinguishing subtle feature differences between sub-classes, but it will also downgrade the prestige of the P-GT. In the following parts of this paper, the evaluation results will be reported regarding these 3 types of classifications when applicable.

### Accuracy

After the model was trained, it predicted the classification of each image set in the test set and compared it to the P-GT. Since the model predicts not only a class but instead the possibilities of being each class, the Top-k accuracy of the model was calculated and depicted to stress the model’s uncertain decisions. The top-k accuracy of the model was defined as the number of correctly predicted tooth classes divided by the total amount of the test set, where we treat a prediction as correct if the P-GT classification resides in the top k largest output units. Formally, let $P$ be the set of predicted items, and $G$ be the set of ground-truth items, with ${g}_{i}\in G$ being the ground truth label of the sample $i$. The top-k accuracy can be defined as:

$$Accuracy@k= \frac{\sum _{i=0}^{N}\mathbb{I}(\text{r}\text{a}\text{n}\text{k}\left({g}_{i}, {P}_{i}\right)\le k)}{N},$$

where $\text{r}\text{a}\text{n}\text{k}({G}_{i}, {P}_{i})$ is the rank of ${g}_{i}$ in the predicted set ${P}_{i}$, $\mathbb{I}$ is the indicator function which is 1 if the condition is true (the item is within the top-k), and 0 otherwise, and $N$ is the total number of samples.

To present a more comprehensive view of the model’s decision on each tooth image set, the confusion matrix was calculated to show the correlation between P-GT and model prediction of each class.

### Per-class analysis

The average per-class sensitivity and specificity were calculated with the following definition:

$$\text{S}\text{e}\text{n}\text{s}\text{i}\text{t}\text{i}\text{v}\text{i}\text{t}\text{y}=\sum _{\text{c}=0}^{{\text{N}}_{\text{c}\text{l}\text{s}}}\frac{\text{T}{\text{P}}_{\text{c}}}{\text{T}{\text{P}}_{\text{c}}+\text{F}{\text{N}}_{\text{c}}}/{\text{N}}_{\text{c}\text{l}\text{s}}$$

$$\text{S}\text{p}\text{e}\text{c}\text{i}\text{f}\text{i}\text{c}\text{i}\text{t}\text{y}=\sum _{\text{c}=0}^{{\text{N}}_{\text{c}\text{l}\text{s}}}\frac{{\text{T}\text{N}}_{\text{c}}}{{\text{T}\text{N}}_{\text{c}}+{\text{F}\text{P}}_{\text{c}}}/{\text{N}}_{\text{c}\text{l}\text{s}}$$

Where c stands for class and ${N}_{cls}$ is the total number of the classes, TP, TN, FN and FP stands for True positive, True negative, False positive, and False negative, respectively.

In addition, the Receiver Operator Characteristic (ROC) curve for each class and their average were analyzed and plotted, with the Area Under the Curve (AUC) calculated for each ROC curve to give a more precise view of the prediction of each class.

### Feature visualization

Besides, to further explore the relationship between different classes from model’s perspective, a feature visualization on the last layer before the output (the embedding layer, d = 512) was conducted by dimensionality reduction. The layer was chosen because it is an ensemble representation of the extracted features from 6 images in the final feature space. The t-SNE method [25] was used to map the higher dimensional feature vectors to 2 dimensional, and the 2-dimensional features were illustrated regarding the predicted class, which enables visualization of the model’s internal decision rule.

As supplementary to the feature domain visualization, we also plotted the feature responses across the spatial dimensions of the image by obtaining the output feature map of the encoder’s last layer. The heatmap is generated by overlapping the absolute value of the feature channels over the original image, which gives an indication of the magnitude and location of the feature responses. This visualization provides a spatial perspective on the model’s feature extraction process to provide a deeper understanding of the model’s behavior and decision-making process.

### Statistical analysis

To evaluate the agreement between human observations, the Fleiss’ Kappa was calculated between A0-A3 on the raw test set (200 teeth). Considering the voting will filter out data where agreement could not be achieved, the Fleiss’ Kappa was also calculated between A0-A3 only on the test set data (129 teeth) to justify the proposed voting strategy.

In addition, to evaluate the consistency of two observers who collected the image, they labeled the raw test set again after two years of the data collection, using the same software and generated their annotation (B2 and B3), the Fleiss’ Kappa was calculated to evaluate the intra-agreement between the raters.

Moreover, the agreement between the P-GT and the model prediction was accessed using the Cohen’s Kappa.

All of the evaluation metrics were analyzed with Python programming language using mainly NumPy and Scikit-learn packages. The statistical analysis was processed using IBM®SPSS® Statistics 27.0 (IBM Corp., Armonk, NY, USA).

## Results

### Data distribution

Table 1 provides a summary of the data distribution for each class as categorized in the initial direct observation (A0). The dataset contains 650 teeth in total, and is well-balanced across classes, with approximately 50 teeth per class for the 16-class classification.

**Table 1:** Class distribution of the dataset

16-type	Class	U1	U2	U3	L1	L2	L3	U4	U5	L4	L5	U6	U7	U8	L6	L7	L8
	**Count**	55	52	50	53	53	54	50	54	52	57	63	49	51	54	45	58
**6-type**	**Class**	**UA**			**LA**			**UP**		**LP**		**UM**			**LM**		
	**Count**	157			160			104		109		163			157		
**3-type**	**Class**	**A**						**P**				**M**					
	**Count**	317						213				320					

### Classification accuracies

The proposed model achieved a classification accuracy (Top-1 accuracy) of 60.47%, 87.60%, and 99.22% for the 16, 3, and 3-type classifications, respectively. The Top-1 to Top-3 accuracies are shown in Table 2. The classification accuracies increased with larger k, where the model achieved Top-3 accuracies of 90.70%, 97.67%, and 100% for the 3 types of classifications, indicating the model performs well to some degree. The average sensitivities and specificities for each class regarding different grouping types are described in Table 3. The model achieved average sensitivities of 58.30%, 87.66%, and 99.31% for the 16, 6, and 3-type classifications, with specificities of more than 95% in all 3 grouping types.

**Table 2:** Top-k accuracies

Class	Top-1	Top-2	Top-3
16-type	60.47%	82.95%	90.70%
6-type	87.60%	94.57%	97.67%
3-type	99.22%	100%	100%

**Table 3:** Average sensitivity and specificity

Class	Sensitivity	Specificity
16-type	58.30%	97.38%
6-type	87.66%	97.58%
3-type	99.31%	95.57%

### Confusion matrix

**Fig. 3:**
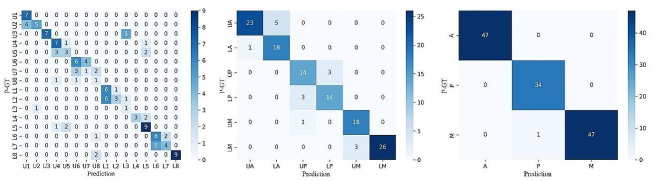
The confusion matrices: it provides a more in-depth description of the model’s prediction, with the number in each cell representing the number of teeth that being categorized in to the corresponding classes

The confusion matrices (Fig. 3) provide a more in-depth description of the model’s prediction, with the number in each cell representing the number of teeth that were being categorized into the corresponding classes. The more data reside in the diagonal line of the matrix, the better model’s prediction resembles P-GT. In our experiment, most of the data were located in the diagonal line of the 3 subfigures of Fig. 3. From Fig. 3, one can see that the outliner data typically resides in the adjacent tooth or its counterpart on the other jaw (e.g., U3 and L3). Furthermore, the number of outliner data decreased with larger classification granularity, i.e., 6-type and 3-type classifications.

### ROC curve and AUC

**Fig. 4:**
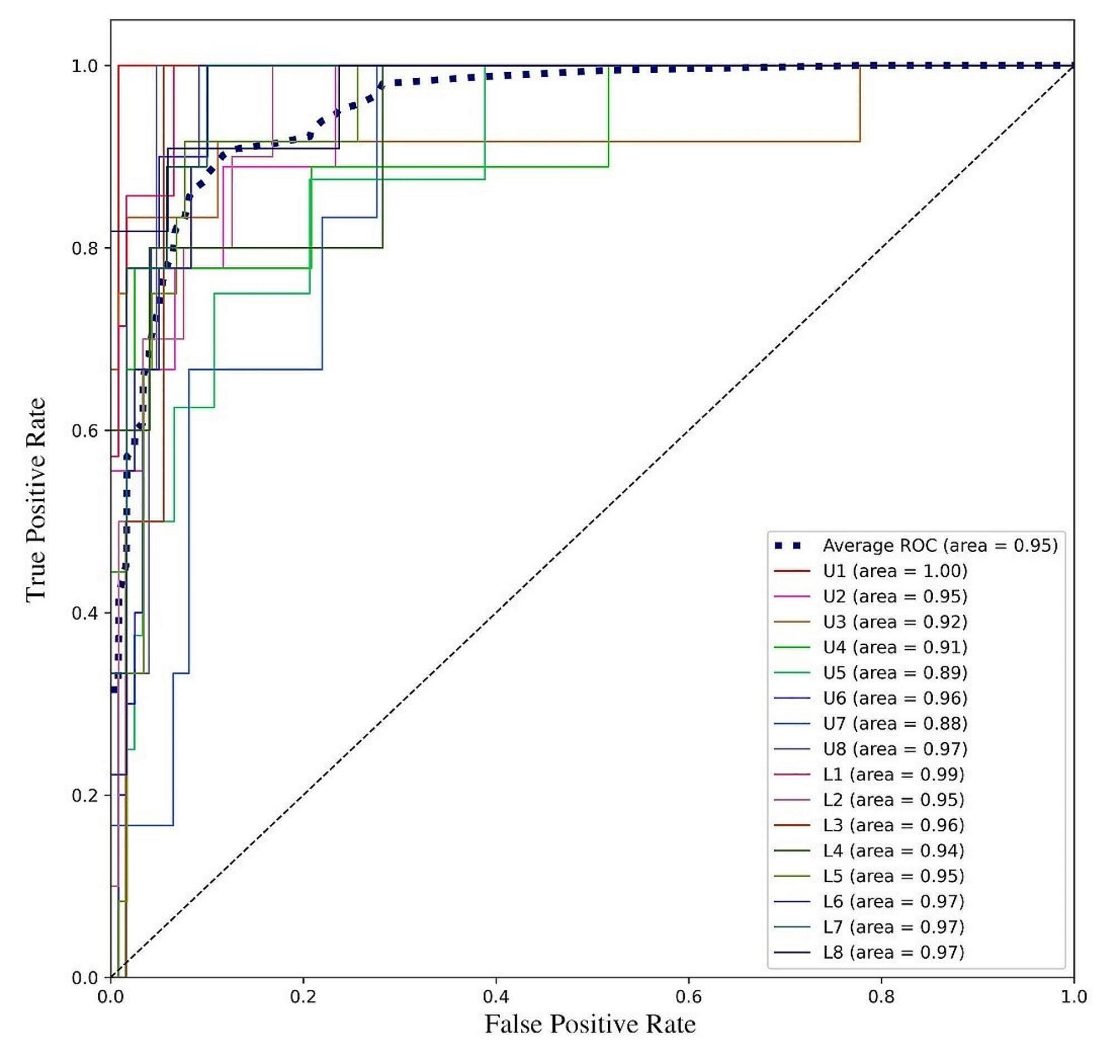
ROC curve and AUC. The ROC curves for each class were plotted in solid lines with different colors, the average ROC curve were plotted as dotted line. The AUCs for each curve were shown in the figure legend

The ROC curves for each class as well as the average ROC curve are plotted in Fig. 4, with AUCs shown in figure legend. The model achieved an average AUC of 0.95 with most of the AUCs exceeding 0.9 for each class.

### Feature visualization

**Fig. 5:**
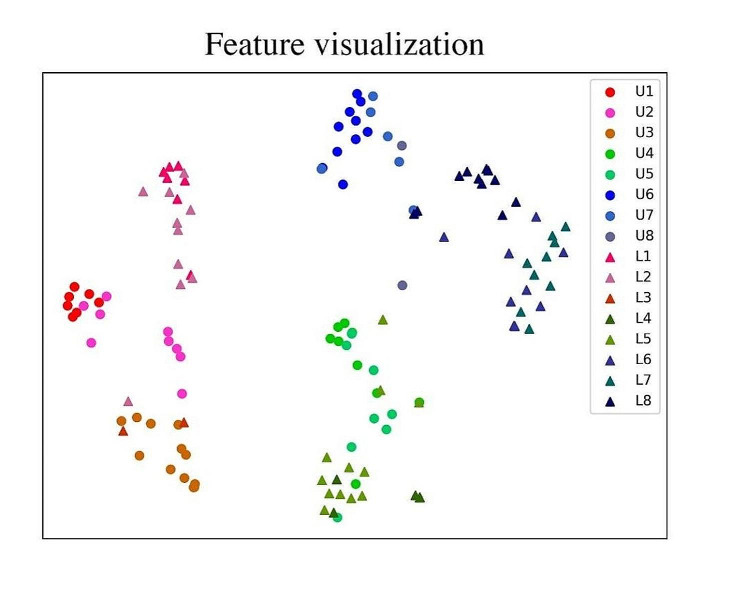
Feature visualization of the last layer before the output by dimensionality reduction. Different colors were given to different classes on P-GT, of which, the anterior teeth, premolar and molar were given reddish, greenish and bluish colors, respective

Figure 5 shows the feature visualization of the embedding layer by dimensionality reduction. A larger inter-class distance with a smaller intra-class distance indicates better performance of the model in feature representation by distinguishing different classes and grouping the same classes. In Fig. 5, different colors are given to different classes of the 16-type classification by the P-GT. To better illustrate the grouping of different super-categories, the anterior teeth (A), premolar (P), and molar (M) are given reddish, greenish, and bluish colors, respectively. In addition, the upper (U) and lower (L) teeth are accordingly plotted in circular and triangular shapes. The model did a good job in separating A, P, and M, it also performed decently in differentiating U and L, however, inaccuracies can be observed in teeth with similar anatomical features, such as L1 and L2.

**Fig. 6:**
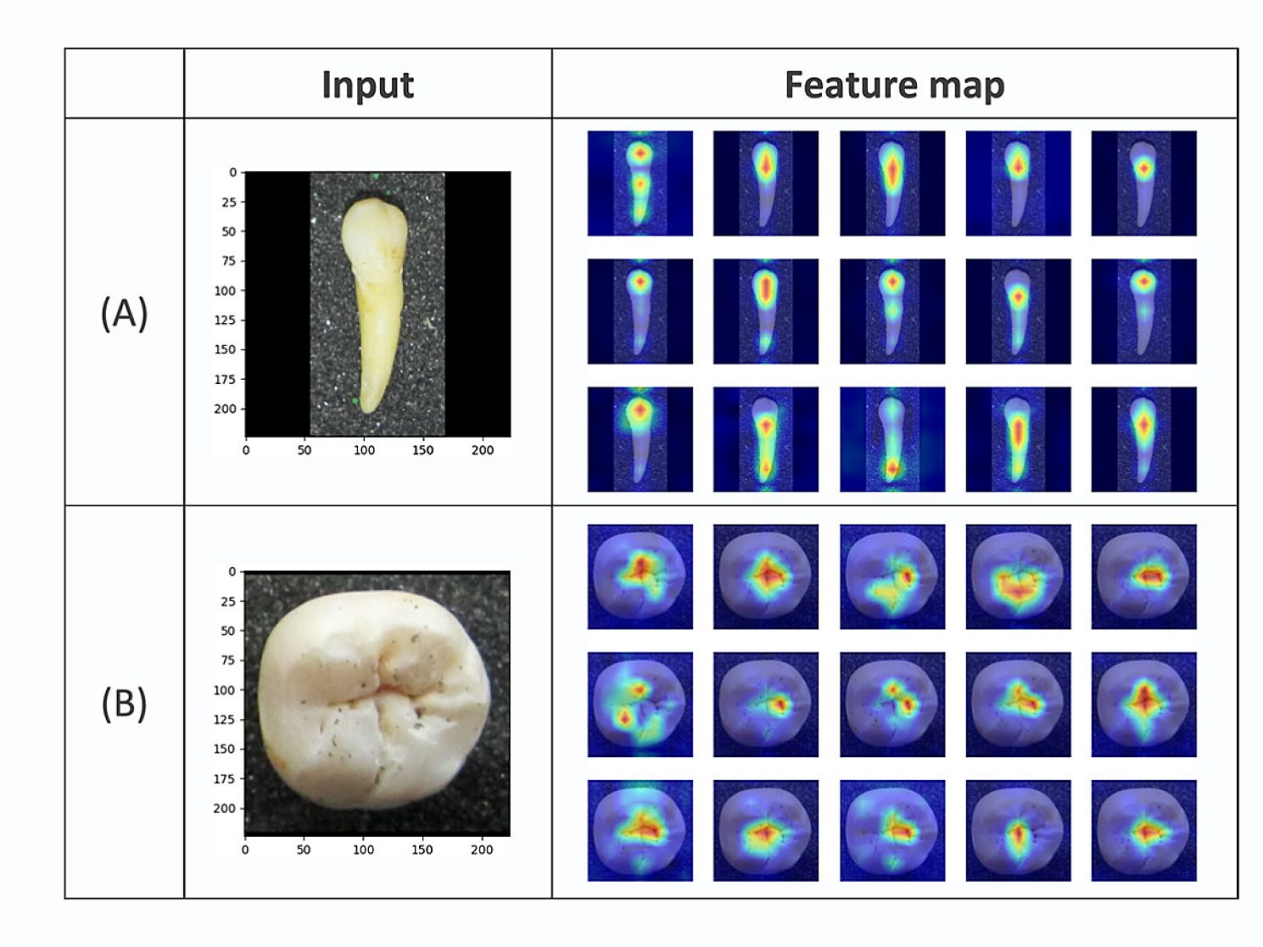
Spatial view of the feature representation from the encoder’s last layer. The heatmap is generated by overlapping the absolute value of the first 15 feature channels over the original image. Reddish regions indicate high activation, while bluish regions indicate low activation

In addition to visualizing the aggregated feature from 1-dimensional feature space, we also plot samples from feature maps of the encoder network in Fig. 6. The encoder model output features of hundreds of channels at its last layer, from which we plot only the first 15 for clarity. This visualization offers a spatial view of the feature representation and shows how the model processes information at the pixel level. By examining Fig. 6, we can identify which regions of the image are most important for the model’s decision-making process and to gain insights into the model’s feature extraction process. As shown in the figure, the model focusses primarily on anatomical structures such as root apices, cervical margin, and the cusps and fissures of the crown, with different channels focusing on different aspect of the image. Overall, this visualization provides valuable insights into the model’s feature extraction process and demonstrates its ability to effectively identify the tooth, rather than relying on irrelevant details or backgrounds.

### Kappa statistics

The results of the Fleiss’ Kappa between the A0 – A3 indexed by raw test set/test set are shown in Table 4. The value under 16-type classification was in 0.4–0.6 intervals indicating moderate agreement, and the value under 6 and 3-type classifications were under 0.6–0.8 and 0.8-1.0 intervals, indicating substantial and almost perfect agreement [26]. We found that all Fleiss’ Kappa in 3 types of classifications increased after filtering, indicating enhanced agreement, which reflects the rationality of the voting strategy.

**Table 4:** The agreement among A0, A1, A2, and A3 indexed by raw test set and resulting test set

Class	Raw test set		Test set	
	Fleiss’ Kappa	95% CI	Fleiss’ Kappa	95% CI
**16-type**	0.441	0.425–0.456	0.571	0.552–0.590
**6-type**	0.688	0.662–0.715	0.781	0.749–0.813
**3-type**	0.924	0.882–0.966	0.945	0.895–0.995

In addition, the Fleiss’ Kappa between the A2 and B2, A3 and B3 indexed by raw test set are shown in supplementary Table ST4, the results are close to that of refined test set, indicating moderate to good intra-observer agreement, this consistency supports the reliability of the labeling process.

The Cohen’s Kappa between P-GT and model prediction are shown in Table 5. Kappa in 16-type is 0.577, indicating moderate agreement. Kappa in 6-type is 0.850 and Kappa in 3-type is 0.988, indicating almost perfect agreement [26].

**Table 5:** Agreement between P-GT and the model prediction using Cohen’s Kappa

Class	Cohen’s Kappa	95%CI
**16-type**	0.577	0.489–0.665
**6-type**	0.850	0.781–0.919
**3-type**	0.988	0.964-1

## Discussion

In this study, we proposed a deep learning model to classify isolated tooth through natural images. Our model was able to accurately classify isolated tooth and performed well under three kinds of classification conditions, achieving accuracies of 60.47%, 87.60%, and 99.22% in 16, 6, and 3-type classifications, respectively.

The proposed deep learning model entailed the usage of ConvNeXt [23] as its feature extraction module, which is one of the most state-of-the-art classification models with sophisticated design that achieved top accuracies in various classification tasks. We also implemented an attention-based module to fuse the extracted features and generate the final prediction. The Transformer, as well as its multi-head attention mechanism, has achieved tremendous success in nearly all of the nowadays machine learning fields [24], it is especially good at correlating and exploring the relationship between features, which is crucial for image set classification tasks that involve multiple views or instances of the same object. We believe this combination provides a strong baseline for our problem. The experiment also showed that the selected model is one of the best architectures for our task (Refer to the supplementary materials).

It has to be noted that the Top-3 accuracy is a more meaningful metric when there are numerous classes in a large dataset. Therefore, it is difficult to use top-3 accuracy as a sole indicator of the model’s performance. The Top-3 accuracies of more than 90% in all classification types indicate the model performs well in generating fuzzy results of visually similar types. The confusion matrix, together with the feature visualization, provides more insights into the model’s decision-making process. The model did well at separating teeth super-groups (e.g., A, P, and M or U and L), and the mistakes were mostly in teeth with similar anatomical structures, typically adjacent teeth (e.g., 4 and 5), which resembled human behavior. An average AUC of 0.95 as well as the high specificity shows the model can generate decent possibilities for each class.

Altogether, the results show that the model can serve as a potential assistive tool in identifying the isolated tooth, which has an enormous application potentiality in dental education. In the future, accuracy will further improve if a larger dataset with better annotation quality is obtained, and our model can be further developed into software for the teaching of teeth identification. Actually, students are passive in the learning process in most cases [27]. Many studies have shown that digital dentistry provides students with useful adjunctive teaching tools for learning dental anatomy [28–30]. Digital dentistry can not only improve the accuracy of students’ identifying teeth but also helps to improve students’ motivation for autonomous learning, and the students indicate that they prefer this teaching method [30, 31]. In situations where a student or nurse/internee faces difficulty in accurately identifying a tooth, they have the option to upload a photo for classification using our model. This process can be performed solely based on the image, eliminating the need for professional assistance. Notably, a sensitivity of 58.3% for 16-type classification indicates that when interpreting the classification result solely by the top-1 probability score, the model’s performance is moderate. However, when similar tooth types are considered, the 6-type and 3-type classification result is more trustworthy. Furthermore, our model provides a probability prediction for each classification, rather than a definitive result. This feature makes it an ideal assistive tool for the teeth classification task, allowing the operator to make the informed decisions. In addition, the practicality of the deep learning model, which is able to classify isolated human teeth from a series of images, could become a valuable tool in forensic dentistry [32], where it could make an important contribution to other methods of reconstructing dental patterns in the identification of human remains.

Moreover, classification model is the foundation for numerous high-level AI applications, it is the backbone of the detection and segmentation models [33], and the base for the generative adversarial networks [34]. The feasibility of tooth type classification using this method opens up avenues for future development of more deep-learning-based tools, particularly generative models, and holds immense potential for practical applications.

### Comparison with prior works

The identification of teeth has been extensively studied in several specific types of dental images, such as panoramic radiographs [20, 35, 36], CBCTs [18, 37, 38], and intra-oral scans [39, 40] where high classification accuracies could be achieved because these kinds of images have fewer pattern variations and clearer adjacent and occlusal relationships between teeth, which is a very important feature for tooth classification. However, in our case, the aforementioned advantages don’t exist. To our knowledge, no previous study has classified real human teeth specimens through photographs. The most similar work is from Miki et al. [22] and Li et al. [21], where they classified the single tooth 3 dimensional volumes cropped from CBCTs. Miki et al. [22] achieved an accuracy of 77.4% under 7-type classification and they further improved it to 88.8% with various data-augmentation techniques [41]. Li et al. [21] achieved an overall classification accuracy of 87.0 under 4-type classification. Compared to the photograph, CBCT crop provides more information about a single tooth: The 3-dimensional shape is available, rather than a 2-dimensional projection; The root canal system, which is also a tooth anatomical characteristic is visible; In addition, the alveolar bone shape and adjacent teeth image inside the cropped region of interest can be utilized as an additional feature in helping determine aimed tooth category. What’s most important, in all other tooth classification studies, the ground-truth (GT) category could be obtained based on various image features, especially the relative tooth position. In our case, all teeth were teaching specimens, the abrasion as well as the lacking of relative teeth positional information makes it impossible to obtain the ground truth. The aforementioned difficulties make our goal an extremely challenging task. However, the study’s findings are still valuable because the single tooth without adjacent teeth nor any type of radiograph is a very common situation in forensic, disaster, and teaching scenarios. Therefore, the results of this study have important research and application value.

To encounter the unknown GT problem, we came up with a voting strategy to generate P-GT based on different human observations, which is based on the assumption that the reliability of the observation differs from the experience of the observers as well as the way they observe the teeth. More weight was given to more reliable observations (A0 and A1), and fewer weight were given to less experienced readers while they observe the teeth from images (A2 and A3). We have empirically chosen the weights so that a P-GT classification will be generated only if one of the following conditions were met: (1) At least the 2 reliable observations agree in the same classification; (2) The 2 reliable observations disagree, but one of them agree with the other 2 less reliable observations. If none of the above conditions were met, the data will be moved out of the dataset. In this way, we largely improved the reliability of the resulting dataset. The voting-based dataset correction was only applied to the test set because, in our practice, we found this procedure filters out more than 30% of the data (71/200) which will largely decrease the data size of the training set if applied. In addition, considering the dataset was labeled as a maximum likelihood estimation of the GT, the label will still be beneficial to the model training by providing a rough gradient direction. The difficult cases can be seen as a regularization of the model to improve its robustness.

### Limitations

Our study has several limitations to be reported: First of all, the train and test sets were both labeled by experienced observers based on tooth anatomy, which was an estimation of the real category, especially when considering that the two observers have a lower expertise level than more experienced teachers or clinicians. Although the test set was later refined and selected to improve its validity, the P-GT is still a compromise to the unknown GT. To overcome these limitations, we took several measures to ensure data accuracy, and to minimize the risk of estimation errors, the strict comparison of the two observers results and the involvement of the third expert should largely improve the reliability of the label, and the P-GT can be seen as a reliable estimation, given that the variability of the tooth anatomy and lack of contextual information make it nearly impossible to accurately identify the tooth type for all instances. Secondly, even if we collected a total of 850 teeth, considering the teeth were classified into 16 categories, the data size for each category is still limited. The best machine learning model can only be trained out of a large number of high-fidelity data. Admittedly, the limitation of data size affects the generalizability and robustness of the model. The limited dataset for each category may further impact the accuracy of identification, especially for visually similar category. Furthermore, the fact that our dataset was prepared with a uniform background and lighting (refer to supplementary Figure SF1) may exacerbate the model’s sensitivity to image variations, robustness to noise, and potential biases. However, in clinical scenarios, it is both impractical and immoral to extract intact teeth except for some rare cases, and the extracted intact teeth are usually limited to the wisdom teeth and premolar, which makes it extremely difficult to collect such a large number of extracted intact teeth with all categories. Additionally, our model lacks external validation on an independent dataset, which is crucial for assessing the generalizability of the model beyond the dataset used for training and testing. It will be one of our future goals to further improve and evaluate this model when larger and more diverse datasets become available. In this study, the dataset was built by taking images of teaching specimens, which are in relatively large quantity, good quality, and have a balanced per-class data size (refer to Table 1). Although the teeth still suffered from non-standard anatomies such as abrasion and caries, it is the best data quality we can get for now, and we adopted several strategies to improve the reliability of the human annotation. We believe that our efforts have provided a reasonable level of confidence in the performance of our model.

## Conclusions

In conclusion, our model is effective in classifying isolated human tooth through a set of images. This study is the first deep learning model that classifies human tooth specimens through natural images. It has the ability to classify isolated human tooth and can serve as an assistive tool for teeth identification teaching in the future. The proposed method has potential applicability in various fields such as teaching, forensic dentistry, and archaeology.

### Electronic supplementary material

Below is the link to the electronic supplementary material.

Supplementary Material 1

## Data Availability

No datasets were generated or analysed during the current study.

## Abbreviations

CBCT: Cone-beam Computed Tomography
FDI: Fédération Dentaire Internationale
P-GT: Pseudo-ground-truth
GT: Ground-truth
ROC: Receiver Operator Characteristic
AUC: Area Under the Curve
A: Anterior teeth
P: Premolar
M: Molar
U: Upper teeth
L: Lower teeth
