# Supplementary material for: Application of deep learning in isolated tooth identification
Source: BMC Oral Health. 2024 May 9;24:500. doi: 10.1186/s12903-024-04274-x (PMC11080190; doi:10.1186/s12903-024-04274-x)
Supplement: Supplementary file 1 — Supplementary Material 1 [file 12903_2024_4274_MOESM1_ESM.docx]

## Supplementary materials

### **Data distribution and data samples**

In this study, two distinct background colors were included in the dataset, namely black and green. To give reader a better understanding of the dataset, we report the exact teeth number used for train set and test set in Table ST1.

**Table ST1.** Exact teeth number used for train set and test set.

| **Train set** | | **Test set** | |
| --- | --- | --- | --- |
| **Black** | **Green** | **Black** | **Green** |
| 223 | 427 | 98 | 102 |

To further demonstrate the details of teeth conditions and show case the dataset samples, parts of data from the train set and test set including black and green background are randomly selected to be shown in Figure SF1 (the images were resized and stretched to fill the canvas for better illustration). Each subplot of Figure SF1 contains fifty teeth. Image samples of the entire dataset will be available upon request.

| 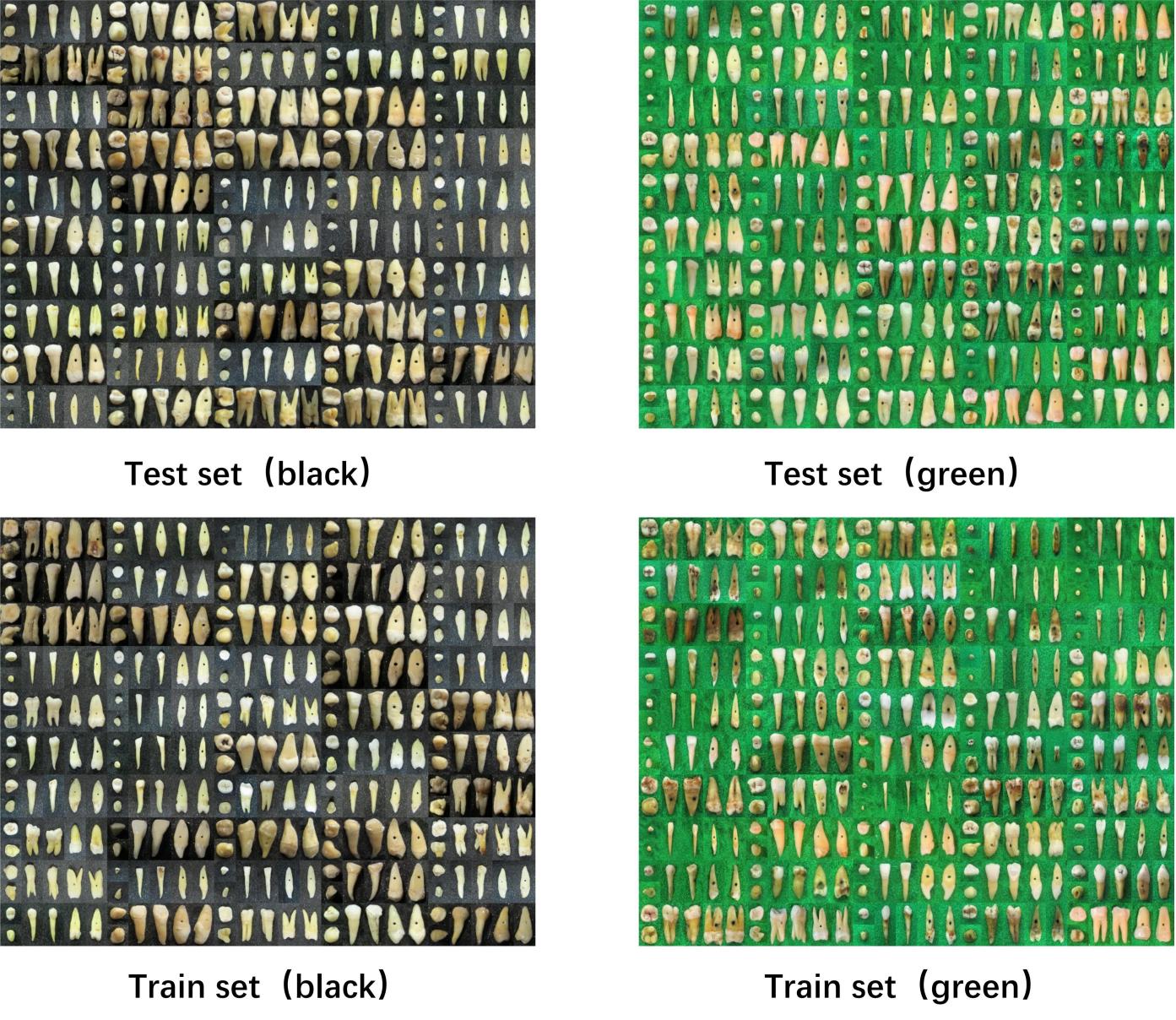  **Figure SF1.** Parts of data samples from the train set and test set including black and green background. Each subplot contains randomly selected fifty teeth to demonstrate the details of teeth conditions and images. |
| --- |

### **Experiments on model design**

**Model setup**

The proposed deep learning model in our study mainly consists of a feature extraction network (backbone) to extract features from each image, then the features will be fed into an encoder which will merge the features and made prediction out of them. A more generalized topology of the model can be depicted as Figure SF2.

| 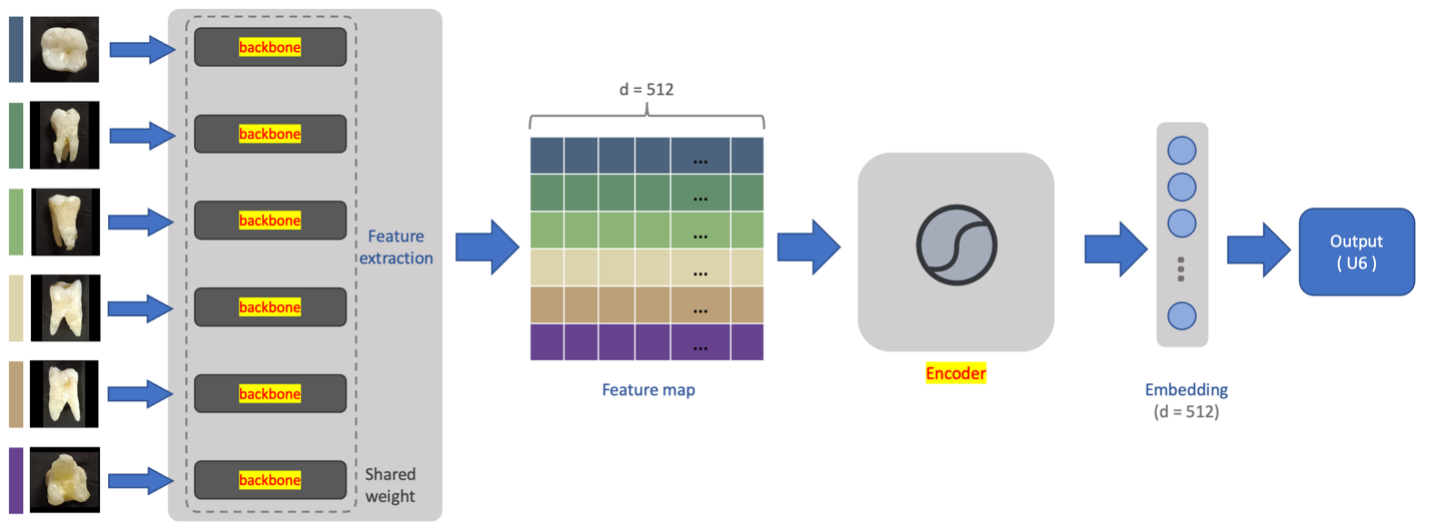  **Figure SF2.** General topology of the model. The model consists of a backbone to extract image feature and an encoder to fuse features and to make prediction. |
| --- |

To justify the selected backbone (ConvNeXt-S) and encoder (Transformer attention encoder), we propose to train and evaluate different models by substituting the backbone with the famous ResNet50 and using fully-connected network with skip connections (FC encoder, Refer to Figure SF3) as the encoder.

Three models using different configurations were proposed, specifically: model-1 use ConvNext-S backbone and attention encoder, which is the one that we adopted in the main article; model-2 use ResNet-50 backbone and attention encoder; model-3 use ResNet-50 backbone and FC encoder (Refer to Table ST2 for an overview of the settings). All 3 models were trained with the same configurations that was described in the main article, and were then evaluated on the P-GT.

| 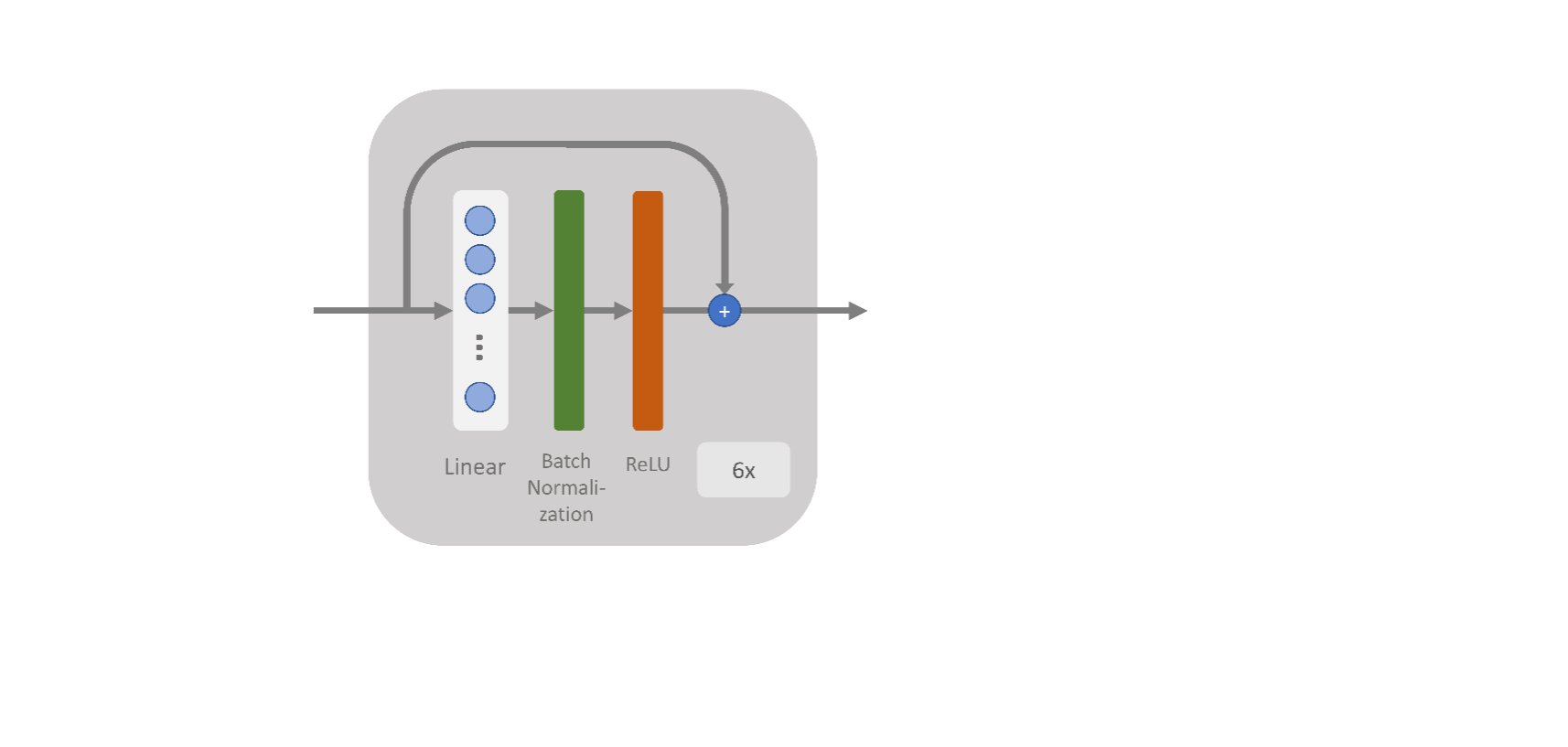  **Figure SF3.** The FC encoder. The fully-connected encoder is made up of 6 identical building blocks, with each being constituted of a linear layer, a batch normalization layer and a ReLU non-linearity layer, together with the skip connection to ease the gradient vanishing problem. |
| --- |

**Table ST2.** Different model setup.

| Name | Backbone | Encoder |
| --- | --- | --- |
| Model-1 | ConvNeXt-S | Attention encoder |
| Model-2 | ResNet-50 | Attention encoder |
| Model-3 | ResNet-50 | FC encoder |

**Results**

The Top-5 accuracies for 16-type classification were reported in Table ST3, the model-1 gets consistently higher accuracies over the other 2 models. The model-2 and model-3 has the same Top-1 accuracy but model-2 performs better in other evaluation levels, which indicates that, comparing to the model-3, it generated more reasonable probabilities for each class.

**Table ST3.** Top-k accuracies for the 3 models

| Name | Top-1 | Top-2 | Top-3 | Top-4 | Top-5 |
| --- | --- | --- | --- | --- | --- |
| Model-1 | 60.47% | 82.95% | 90.70% | 96.90% | 96.90% |
| Model-2 | 46.51% | 72.87% | 81.40% | 88.37% | 94.57% |
| Model-3 | 46.51% | 63.57% | 75.19% | 79.07% | 83.72% |

The ROC curves for each model were plotted in Figure SF4. The model-1, model-2 and model-3 get an average AUC of 0.95, 0.94 and 0.85, respectively. The results show that the model-1 and model-2 does better in generating prediction probabilities than the model-3.

| Model 1  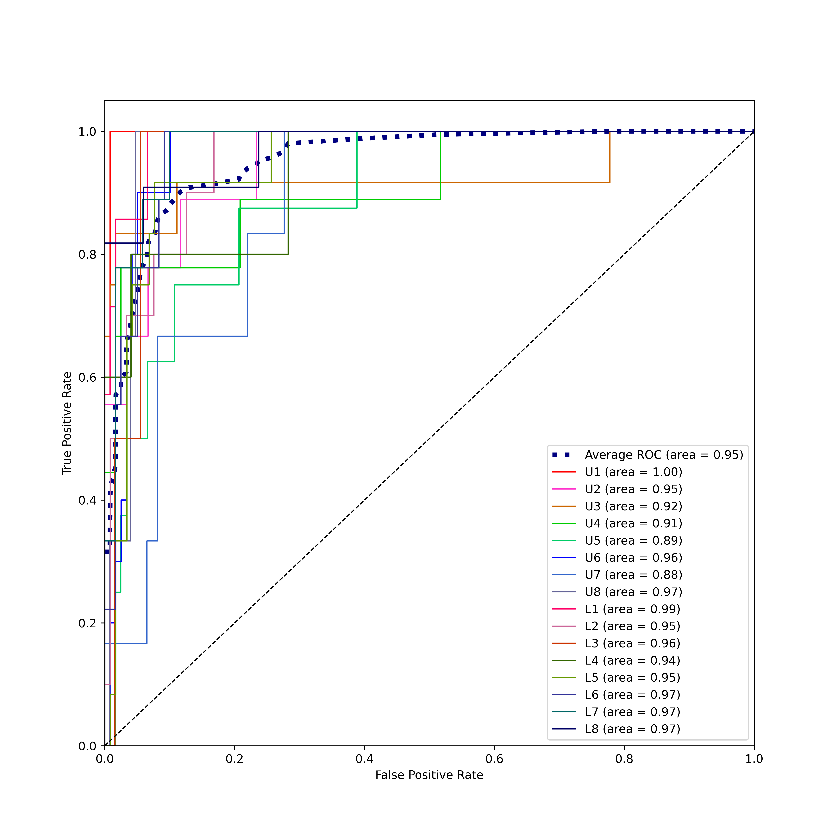 | Model 2  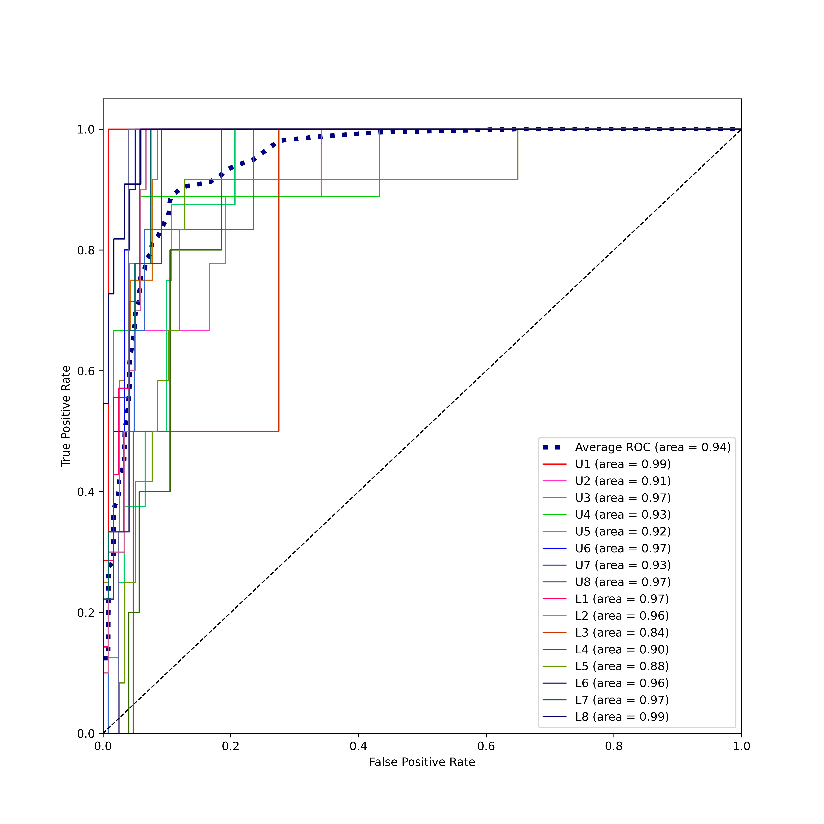 |
| --- | --- |
| Model 3  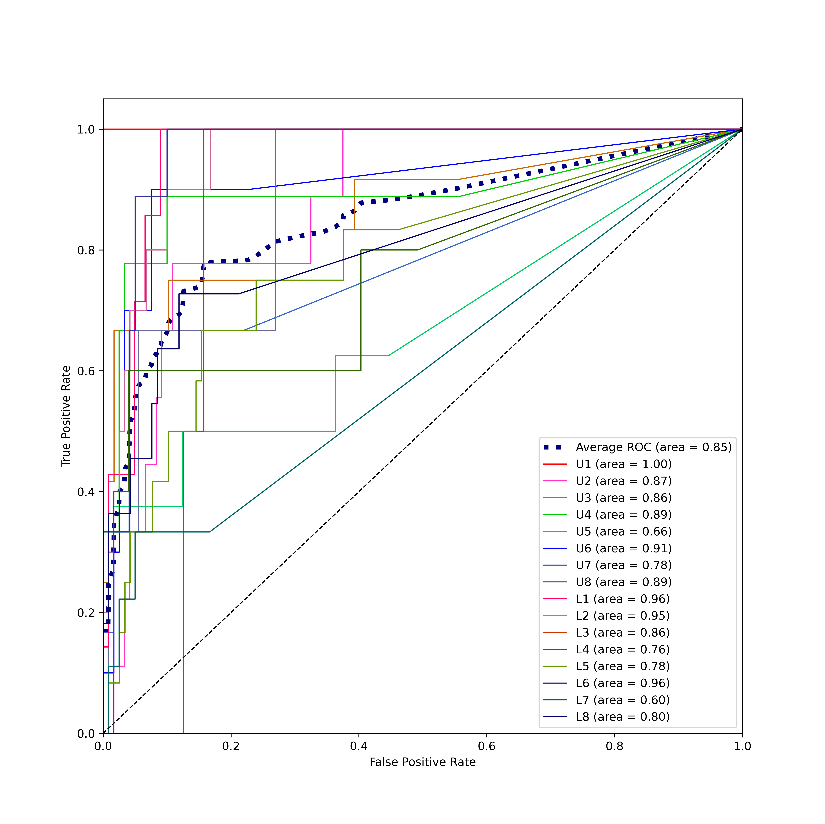 | **Figure SF4.** ROC curves for each model. AUC were calculated and depicted in the figure legends. |

Altogether, our results prove that with state-of-the-art topology design, the proposed deep learning model has the optimal setup for our task.

1. **Intra-observer agreement statistics**

To ensure the consistency of two observers who collected the image, the Fleiss' Kappa was calculated to evaluate the intra-agreement. The two observers labeled the raw test set again after two years of the data collection, using the same software and generated their annotation (B2 and B3). The values of the 3 types of classifications are reported in Table ST4. The value under 16-type classification were mostly in 0.4-0.6 intervals, indicating moderate agreement, and the value under 6 and 3-type classifications were under 0.6-0.8 and 0.8-1.0 intervals, indicating substantial and almost perfect agreement. The results justify the reliability of the labeling process.

**Table ST4.** The agreement between A2 and B2, A3 and B3, indexed by raw test set.

| **Class** | **The agreement between A2 and B2** | | **The agreement between A3 and B3** | |
| --- | --- | --- | --- | --- |
|  | **Fleiss' Kappa** | **95% CI** | **Fleiss' Kappa** | **95% CI** |
| **16-type** | 0.565 | 0.528-0.603 | 0.601 | 0.563-0.640 |
| **6-type** | 0.745 | 0.682-0.809 | 0.746 | 0.683-0.809 |
| **3-type** | 0.970 | 0.871-1 | 0.932 | 0.833-1 |
